# Editorial: Multi-omics, Epigenomics, and Computational Analysis of Neurodegenerative Disorders

**DOI:** 10.3389/fnins.2022.930425

**Published:** 2022-07-06

**Authors:** Manoj Kumar Jaiswal

**Affiliations:** Department of Psychiatry, Icahn School of Medicine at Mount Sinai, New York, NY, United States

**Keywords:** multi-omics, epigenomics, neurodegeneration, computation, machine learning

With the dawn of multi-omics technologies, integrated with computation and biostatistics, remarkable progress has been made in understanding the pathobiology of neurodegenerative diseases such as Amyotrophic Lateral Sclerosis (ALS), Frontotemporal Dementia (FTD), Alzheimer's disease (AD), Parkinson's Disease (PD), Aging, and others. Multi-omics approaches such as genomic/epigenomic, transcriptomic, proteomic, metabolomics, and miRNAomics, as well as genetic and functional perturbations have changed the experimental modeling of these diseases. For example, in ALS, genomic approaches such as genome wide association studies (GWASs) have led to the discovery of relatively few loci despite 52% heritability estimates (Zhang et al., 2022). However, these changes occur in <10% of ALS patients, and thus, there are likely to be many ALS risk genes yet to be discovered. Similarly, in FTD (Ferrari et al., 2014), AD (Bertram and Tanzi, 2009), PD (Kia et al., 2021), Aging (Walter et al., 2011), and Dementia (Moreno-Grau et al., 2019) GWAS has led to the identification of few candidate genes, novel loci and unique associations, with evidence of disease-associated regulatory changes. Brain region and cell-sub-type-specific (dys)functional multi-omics studies in neurodegenerative diseases, such as RNA-Seq, ChIP-Seq, ATAC-Seq, and Hi-C has fueled genetic discovery in a cell-type dependent manner. For this special Research Topic, we present a compendium of 10 articles, which offer a wide-ranging overview of the different multi-omics pathways and unravel the genomic and transcriptomic alterations in these diseases as well as endeavor to facilitate a better understanding of the mutational landscape of these disorders.

To enable new biomarker discovery in ALS, Dr. Gabriel and colleague examines the whole (unbiased) metabolomics data to study changes in spinal cord regions of two strains of mutant SOD1 mice with fast (129S) or slow (C57BL) disease progression associated with SOD1-G93A ALS transgenic mouse models (Valbuena et al.). The authors show that the C57BL have a more favorable bioenergetic and metabolite profile, including neurotransmitter amino acid metabolism and antioxidant homoeostasis, which were determined to be greatly affected in the thoracic segment. Changes in energy and lipid metabolism were mostly apparent in the lumbar spinal cord and these changes were mainly attributed to background differences between the two strains. Dr. Masashi Aoki's group presents insights into the complexity of multi-omics alteration of motor neurons (MNs) axonal defects in ALS. The authors review the evidence coming from genetic subtypes of ALS and further discuss the potential disease pathways leading to axonal defects (Suzuki et al.). The study by Thompson et al. examines the proteome of ALS and PD patients compared to controls using weighted gene co-expression network analysis (WGCNA) to identify pathways and ontological groupings of interest that differentiate patient groups with particular emphasis on ALS patients. They identify nine co-expression modules, approximately half of which cannot be annotated using Gene Ontology (GO). Among the characterized modules, they found ones associated with intracellular compartments and RNA biology (module 1), the immune system (module 2), which is hypothesized to reflect blood contamination, and axon outgrowth (module 4). They perform paired differential correlation analyses as a more focused correlation approach and find that 11 co-expression modules are altered in ALS, with enrichment in modules 1 and 3, although these modules were without GO enrichment.

In a study on AD, Dr. Andres Kriete's group applied a novel combination of computational strategies to dorsolateral prefrontal cortex RNA-Seq samples from 503 cognitively well-characterized human subjects from the Religious Orders Study/Memory and Aging Project (ROSMAP) and identify 26 distinct modules, encapsulating 4,429 genes (Malamon and Kriete). More specifically, authors organized modules using widely used WGCNA approach and find several gene co-expression modules that become less co-expressed in AD (which they define as “topological erosion”). One of these modules is enriched in genes involved in immune and synaptic system processes. They also report a loss of modular gene expression associated with cognitive decline. Fewer genes remained within statistically preserved modules with the transition from normal cognition through MCI to AD. In addition, they compare SNPs with gene expression from the same donors using expression quantitative trait loci (eQTL) analysis and find that the most significant eQTLs are from the Microtubule Associated Protein Tau (MAPT) locus and the Human Leukocyte Antigen (HLA) complex. Dr. Dhanwani's group carried out a comprehensive study focused on deciphering the role of neuronal antigen specific T cell responses in AD patients and compare these patients with age-matched healthy controls (Dhanwani et al.). The authors have carefully measured T cell responses to several potential antigens and found no differences in the antigen-specific T cell (ASTC) reactivity tested for antigens, relative frequency of major PBMCs subsets, and the expression of genes between AD and healthy controls. Li et al. leveraged several bulk gene expression data sets to identify the association between autophagy-related genes and clinical symptoms of AD using bioinformatics approaches. The study identified 80 autophagy-related genes with differential expression in the brain tissue of patients with AD compared to healthy age-matched control. The expression of a cluster of autophagy-related genes (*n* = 16) correlated with AD clinical symptoms. The authors relate seven autophagy-related proteins that are down-regulated in the brain of AD patients, such as MEF2A and CUX1, with the progression of symptoms of AD patients and focused on the study of MEF2A in detail. A subset of seven autophagy genes were selected as they overlapped two analyzed datasets, and the MEF2A transcription factor was identified as a potential regulator of the expression of the seven genes. MEF2A levels were decreased in AD cases compared to control brain homogenate. Lv et al. describe changes in m6A modifications in the hippocampus of mice harboring a loss-of-function mutation in the gene coding for TYRO Protein Kinase Binding Protein (TYROBP), and serve as a mouse model for Nasu-Hakola disease (NHD). The authors have reported higher levels of total tau, phosphorylated tau, and amyloid β, all of which are correlated with AD and NHD phenotypes. They observe striking reductions in all three RNA methytransferases. Key regulators of the m6A writer machinery (METTL3, METTL14, and WTAP) were also downregulated in terms of relative mRNA and protein levels, contrasting with AD models, while expression of the demethylases FTO and ALKBH5 were largely unchanged.

Tian Tian's group carried out a comprehensive review discussing advances in metabolomics approaches in PD (Zhang et al.). The authors highlight the genetic mutations and mitochondrial dysfunction that occur in patients with mutations and sporadic abnormalities. The authors also described synuclein and parkin gene mutations and functions, as well as concisely recap new metabolomic discoveries in both familial and sporadic PD. Together, this review provides a way forward to advance our current understanding of metabolomics of PD.

David Alan Bennett's group studied four different epigenetic clocks, as calculated on CD4+ cells derived from blood, postmortem DLPFC and PCC samples from participants in the ROSMAP aging study (Grodstein et al.). The authors use Pearson analysis to compare how well four established epigenetic clocks—Horvath, Hannum, PhenoAge, and GrimAge—correlate with chronologic age in ROSMAP subjects with matched CD4+ blood cells from longitudinal blood draws (~7.5 year interval) and matched DLPFC. The main results reported were that DNAm age estimated from brain samples was consistently lower than age at death, whereas the correlation between DNAm age and chronological age was reasonable. Mean clock age was consistently lower than chronologic age in the brain samples. GrimAge correlated best with *r* = 0.92. Epigenetic age modestly correlated with age in matched blood and brain samples (again GrimAge performed best at *r* = 0.76). Finally, in the oldest-old subjects, these correlations were much weaker.

We recognize that a single collection of articles cannot comprehensively cover the entirety of the extremely broad range of topics that characterize such complex and multifactorial conditions such as neurodegeneration, nor the entire gamut of multiomics modalities. The topics addressed, however, help develop a clear idea, not only of what has been accomplished to date by previous studies, but also of the unmet needs future research should focus on. We are confident that the papers assembled in this Research Topic will prove useful in spurring and stimulating the future progress.

## Author Contributions

MKJ conceived, prepared, edited, and approved the article for publication. All contents of this editorial have been solely carried out by MKJ.

## Author Disclaimer

The views expressed in this article are those of the author and do not reflect the official policy or position of the Icahn School of Medicine at Mount Sinai.

## Conflict of Interest

The author declares that the research was conducted in the absence of any commercial or financial relationships that could be construed as a potential conflict of interest.

## Publisher's Note

All claims expressed in this article are solely those of the authors and do not necessarily represent those of their affiliated organizations, or those of the publisher, the editors and the reviewers. Any product that may be evaluated in this article, or claim that may be made by its manufacturer, is not guaranteed or endorsed by the publisher.

## Abbreviations

ALS: amyotrophic lateral sclerosis
AD: Alzheimer's disease
ASTC: antigen-specific T cell
PD: Parkinson's disease
FTD: frontotemporal dementia
DEGs: differentially expressed genes
EHR: electronic health records
GO: gene ontology
GWAS: genome-wide association studies
HLA: human leukocyte antigen
KEGG: Kyoto encyclopedia of genes and genomes
eQTL: expression quantitative trait loci
MAPT: microtubule associated protein tau
MNs: motor neurons
NHD: Nasu-Hakola disease
PCA: principal components analysis
ROSMAP: religious orders study/memory and aging project
SOD1: super oxide dismutase 1
TYROBP: TYRO protein kinase binding protein
WGCNA: weighted gene co-expression network analysis
WGS: whole genome sequencing.
